# Rare Indications of Splenectomy: A Case Series and Literature Review

**DOI:** 10.7759/cureus.95036

**Published:** 2025-10-21

**Authors:** Nivash Maran, Sugumar Chidambaranathan, Krishna Moorthy

**Affiliations:** 1 Surgical Gastroenterology, Madras Medical College, Chennai, IND

**Keywords:** afibrinogenemia, angiosarcoma spleen, atraumatic splenic rupture, pancreatitis, rare indications, ruptured splenic abscess, splenectomy, splenic cyst

## Abstract

Splenectomy is a procedure, most commonly performed for trauma, hematological disorders, and certain malignancies. Rare indications such as congenital bleeding disorders, pancreatitis complications, and splenic cysts may also warrant splenic removal, especially in life-threatening conditions or diagnostically unclear scenarios. In this retrospective descriptive case series, we report cases of six patients who underwent splenectomy for rare indications. The first case involves a 13-year-old boy with congenital afibrinogenemia who presented with signs of acute abdomen and class 3 hypovolemic shock. Computed tomography (CT) imaging of the abdomen and pelvis revealed a large perisplenic hematoma and hemoperitoneum suggestive of spontaneous hemorrhage. Despite fibrinogen replacement therapy, the patient remained hemodynamically unstable, and emergency open splenectomy was performed, which proved to be life-saving. The second and third cases involved middle-aged men with chronic alcoholic pancreatitis who developed large intrasplenic pseudocysts with spontaneous rupture, evidenced by contrast extravasation on CT. Both underwent emergency splenectomy with distal pancreatectomy. Histopathology revealed pseudocysts with no evidence of malignancy. These cases confirm how the anatomical proximity between the pancreatic tail and spleen can facilitate enzymatic injury leading to splenic rupture, an infrequent but dangerous complication of chronic pancreatitis. The remaining two cases were two young female patients who presented with large splenic cysts, and one of them had undergone prior percutaneous catheter drainage and developed infection. Both underwent elective splenectomy due to persistent symptoms and diagnostic uncertainty. Histopathology revealed a benign epithelial cyst in one and a splenic abscess in the other, illustrating the importance of definitive surgical management in selected cystic lesions of the spleen. The final and most aggressive case involved a 44-year-old woman who presented in hemorrhagic shock. Imaging revealed a ruptured splenic mass with multiple lesions in the liver. Emergency splenectomy was performed, and histopathology confirmed primary angiosarcoma of the spleen. Despite surgical intervention, the patient succumbed postoperatively, underscoring the dismal prognosis associated with this rare vascular malignancy. Each case highlights the diagnostic and therapeutic role of splenectomy in atypical scenarios. Awareness of such rare indications can aid timely surgical decision-making when imaging or conservative strategies are inconclusive or ineffective. Though uncommon, conditions such as congenital bleeding disorders, pancreatitis-related complications, and primary benign splenic cyst must be considered in the differential diagnosis of splenic pathology. Splenectomy remains a definitive solution in selected cases.

## Introduction

Splenectomy is a well-established surgical intervention typically indicated for traumatic injury, hematologic disorders such as immune thrombocytopenic purpura (ITP) and hereditary spherocytosis, and certain malignancies involving the spleen [1,2]. In these contexts, splenectomy offers both therapeutic and, at times, diagnostic benefits. However, less frequently encountered scenarios also warrant splenectomy, often presenting as diagnostic dilemmas or as complications requiring urgent surgical management [3].

Among these, rare indications are congenital coagulopathies such as afibrinogenemia, an autosomal recessive inherited condition characterized by absent or severely reduced fibrinogen levels, where spontaneous hemorrhagic events may involve the spleen and become life-threatening [4-7]. Acute or chronic pancreatitis can also result in perisplenic complications, including intrasplenic pseudocyst formation and eventual rupture due to enzymatic digestion or localized vascular injury [8-11]. Furthermore, primary splenic cysts, although mostly benign and asymptomatic, can enlarge, become symptomatic, get infected, or be misdiagnosed as parasitic or neoplastic lesions, thereby necessitating splenectomy [12-14]. Finally, primary splenic angiosarcoma, an extremely rare and aggressive vascular tumor, may present with spontaneous rupture and widespread metastasis, leading to rapid deterioration and death if not promptly addressed [15,16].

Over the past few decades, the overall incidence of splenectomy has declined due to advances in imaging, interventional radiology, and minimally invasive splenic preservation techniques. In resource-limited or emergent settings, radiological uncertainty and clinical instability often necessitate early surgical exploration and splenic removal. This case series describes six patients who underwent splenectomy for such uncommon conditions, highlighting the diverse presentations and emphasizing the importance of considering splenectomy even in non-traditional clinical scenarios. A brief literature review is included to contextualize these cases and underline the diagnostic and therapeutic value of splenectomy in selected patients.

## Case presentation

Case 1: Congenital afibrinogenemia with spontaneous splenic hemorrhage

A 13-year-old boy, with congenital afibrinogenemia diagnosed during childhood, and on irregular treatment and follow-up, presented with sudden-onset left upper quadrant pain, dizziness and abdominal distension for one day. On presentation, he was hypotensive and anemic with elevated prothrombin time and activated partial thromboplastin time (>180 sec) with undetectable serum fibrinogen (Table 1).

**Table 1:** Summary of laboratory investigations PT: Prothrombin time; APTT: Activated Partial Thromboplastin Time; WBC: White Blood Cell.

Cases	Age/Sex	Hemoglobin (g/dl)	WBC count (cells/mm^3^)	Platelets (x10^9^/L)	PT (sec)	APTT (sec)	Fibrinogen (mg/dl)	Amylase (U/L)	Lipase (U/L)
Normal values	-	12 to 16	4000-11000	150-400	11 to 14	25-35	200-400	23-85	0-160
Case 1	13/M	7.4	9800	210	>60	>180	Undetectable	---	---
Case 2	39/M	8.2	12800	330	Normal	Normal	Normal	420	695
Case 3	45/M	7.9	14500	280	Normal	Normal	Normal	385	740
Case 4	25/F	11.2	8700	250	Normal	Normal	Normal	---	---
Case 5	27/F	10.4	16200	290	Normal	Normal	Normal	---	---
Case 6	44/F	5.8	15600	110	Prolonged	Prolonged	---	---	---

**Figure 1:**
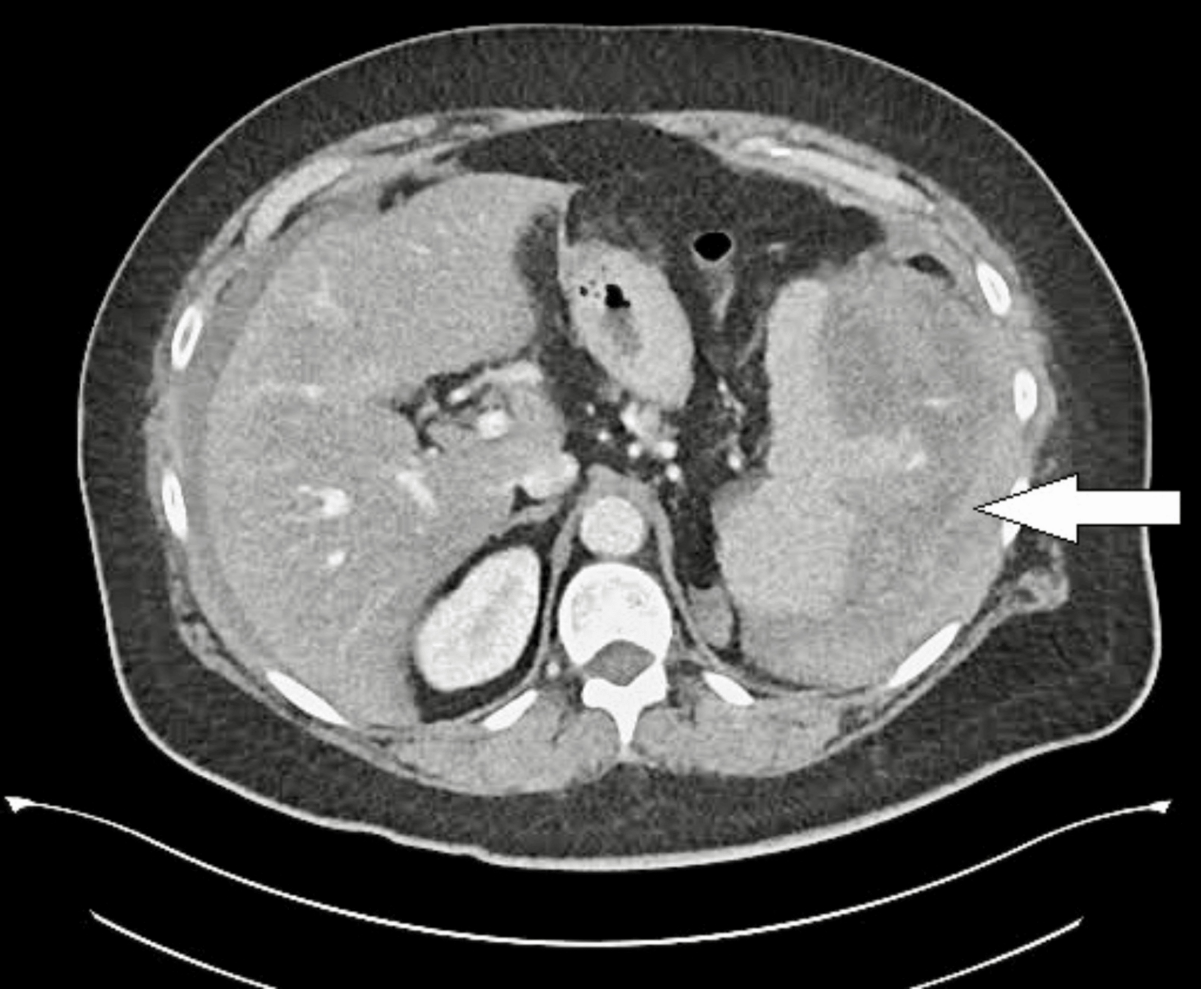
Congenital afibrinogenemia with spontaneous splenic hemorrhage Contrast enhanced CT abdomen and pelvis showing moderate hemoperitoneum with splenic parenchymal rupture. The white arrow indicates the splenic parenchymal rupture.

CT imaging of the abdomen and pelvis revealed a large hypodense collection sized 7x8x5 cm noted around the spleen with moderate hemoperitoneum (Figure 1).

**Figure 2:**
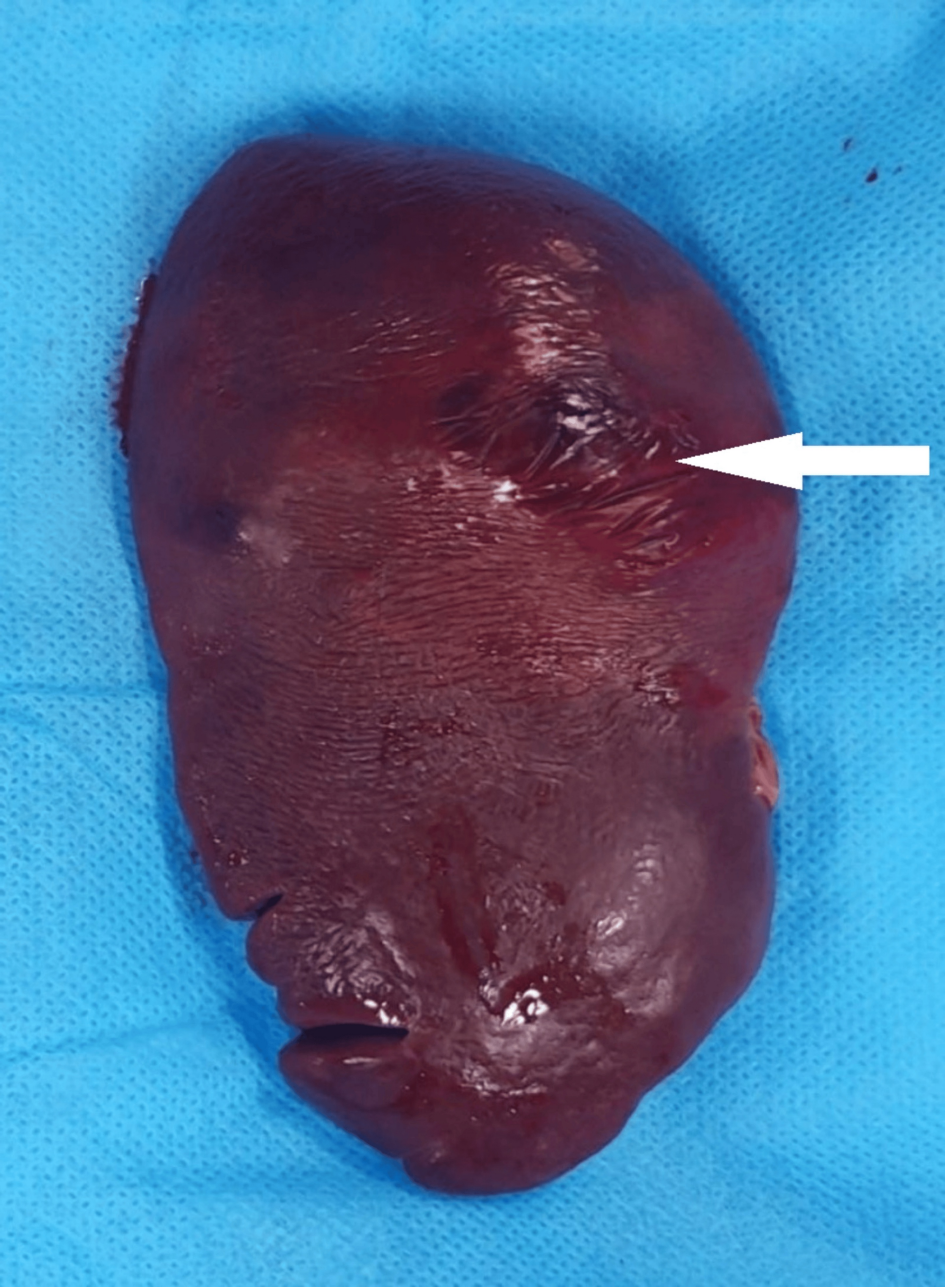
Congenital afibrinogenemia with spontaneous splenic rupture Gross specimen showing splenic parenchymal rupture. The white arrow shows parenchymal laceration.

Even after administration of cryoprecipitate and fibrinogen concentrates, the patient remained hemodynamically unstable. Emergency open splenectomy was performed. Intraoperative findings included two liters of hemoperitoneum and a friable spleen with capsular rupture (Figure 2).

Postoperative recovery was uneventful, and he was advised long-term hematological follow-up and vaccination as per standard guidelines.

Case 2: Rupture of intrasplenic pseudocyst in chronic pancreatitis

**Figure 3:**
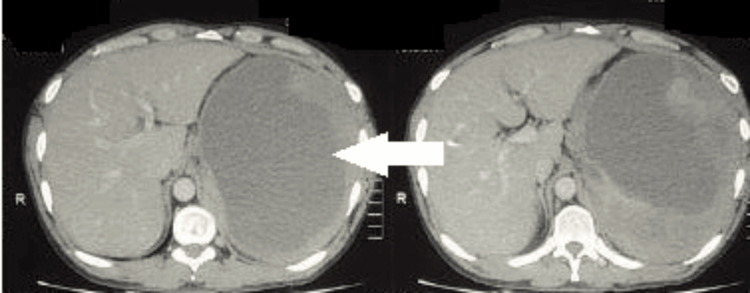
Intrasplenic pseudocyst rupture in chronic pancreatitis Axial CT abdomen showing a large hypodense cystic lesion within the upper pole of the spleen with signs of rupture (white arrow).

A 39-year-old man diagnosed with chronic alcoholic pancreatitis three years ago, presented with abdominal discomfort and early satiety for five days. CT imaging of abdomen and pelvis showed a well defined hypodense cystic lesion of size 10x13x12 cm seen involving upper pole of spleen with active pooling of contrast into the splenic parenchyma and a small pseudocyst 3x2.5 cm in the tail of pancreas with mild hemoperitoneum, suggesting the possibility of rupture (Figure 3).

**Figure 4:**
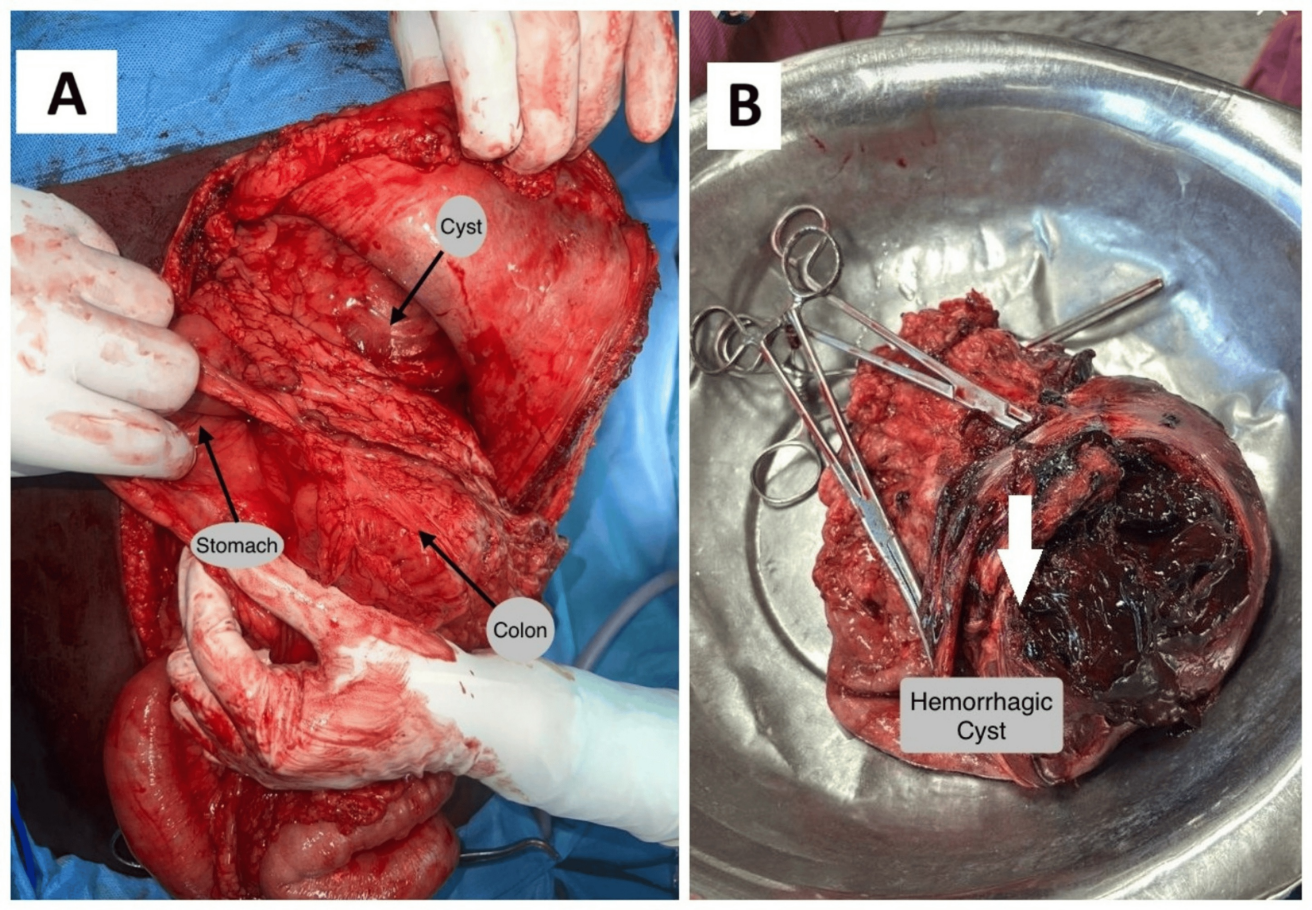
Ruptured splenic pseudocyst (A) Intraoperative picture showing the splenic pseudocyst; (B) Gross specimen of the spleen showing the ruptured hemorrhagic pseudocyst (marked with white arrow).

On presentation he was anemic, resuscitation was done with blood and blood products, and in view of aggravation of symptoms, he underwent emergency open splenectomy with distal pancreatectomy. Intraoperatively, the spleen showed a pseudocyst adjacent to the pancreatic tail, with surrounding fibrosis (Figure 4A).

Figure 4B shows gross specimen showing hemorrhagic cyst. The patient recovered uneventfully.

Case 3: Intrasplenic pseudocyst rupture

**Figure 5:**
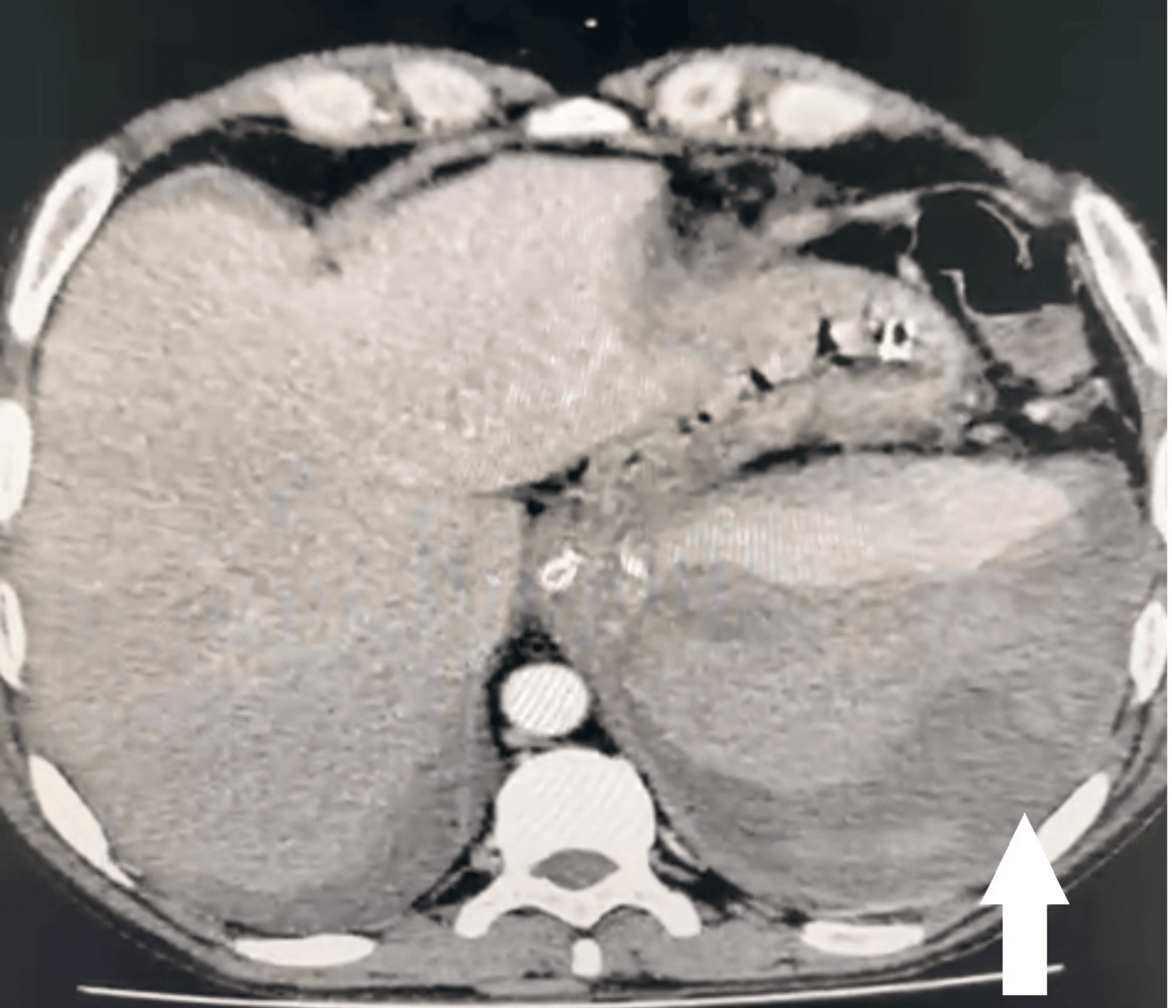
Intrasplenic pseudocyst rupture in chronic pancreatitis Axial CT showing subcapsular splenic hematoma with rupture (white arrow).

A 45-year-old man, a known case of alcohol induced chronic pancreatitis diagnosed one year ago, presented with complaints of diffuse abdominal pain for one day. He was hypotensive and anemic at the time of admission. Contrast enhanced CT abdomen and pelvis showed a large subcapsular collection 12x11x9 cm in the posterior aspect of the spleen tracking along left paracolic gutter with evidence of contrast extravasation near the hilum (Figure 5).

**Figure 6:**
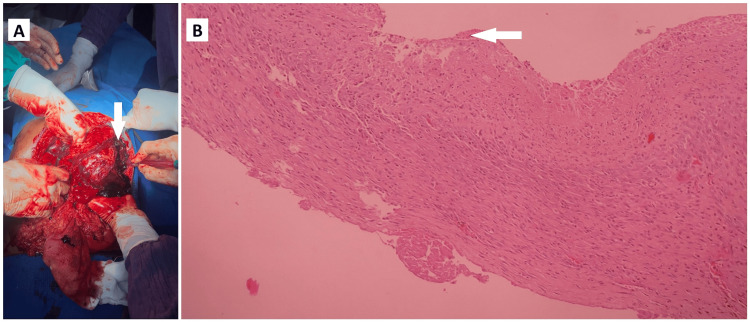
Splenic pseudocyst rupture (A) Intraoperative picture showing splenic parenchymal rupture with hemoperitoneum (white arrow); (B) Histopathology image showing pancreatic parenchyma with pseudocyst without an epithelial lining (white arrow).

He was planned for emergency laparotomy and intraoperative findings were ruptured splenic hematoma with moderate hemoperitoneum (Figure 6A).

He underwent distal pancreatosplenectomy. HIs postoperative period was uneventful. Histopathological examination showed pancreatic parenchyma with pseudocyst (Figure 6B).

Case 4: Large primary splenic cyst

**Figure 7:**
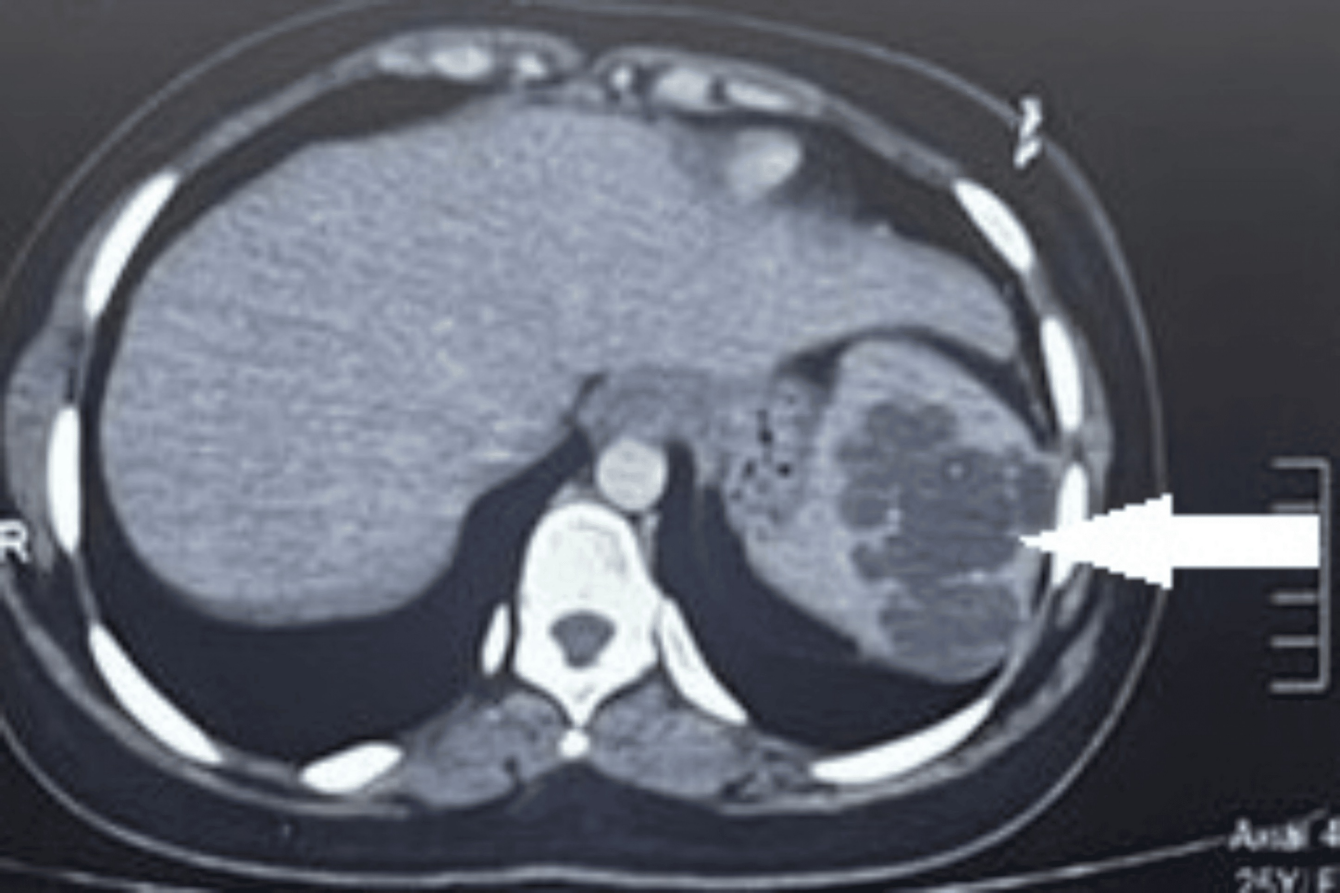
Primary splenic cyst Contrast enhanced CT abdomen image showing a multiloculated cyst in the superior pole of the spleen (white arrow).

A 25-year-old lady presented with left upper quadrant vague abdomen pain for four months. CT imaging revealed a 6.9x3.9 cm multiloculated cystic lesion in the superior pole of spleen with thin non-enhancing septations with the possibility of a splenic cyst (Figure 7).

**Figure 8:**
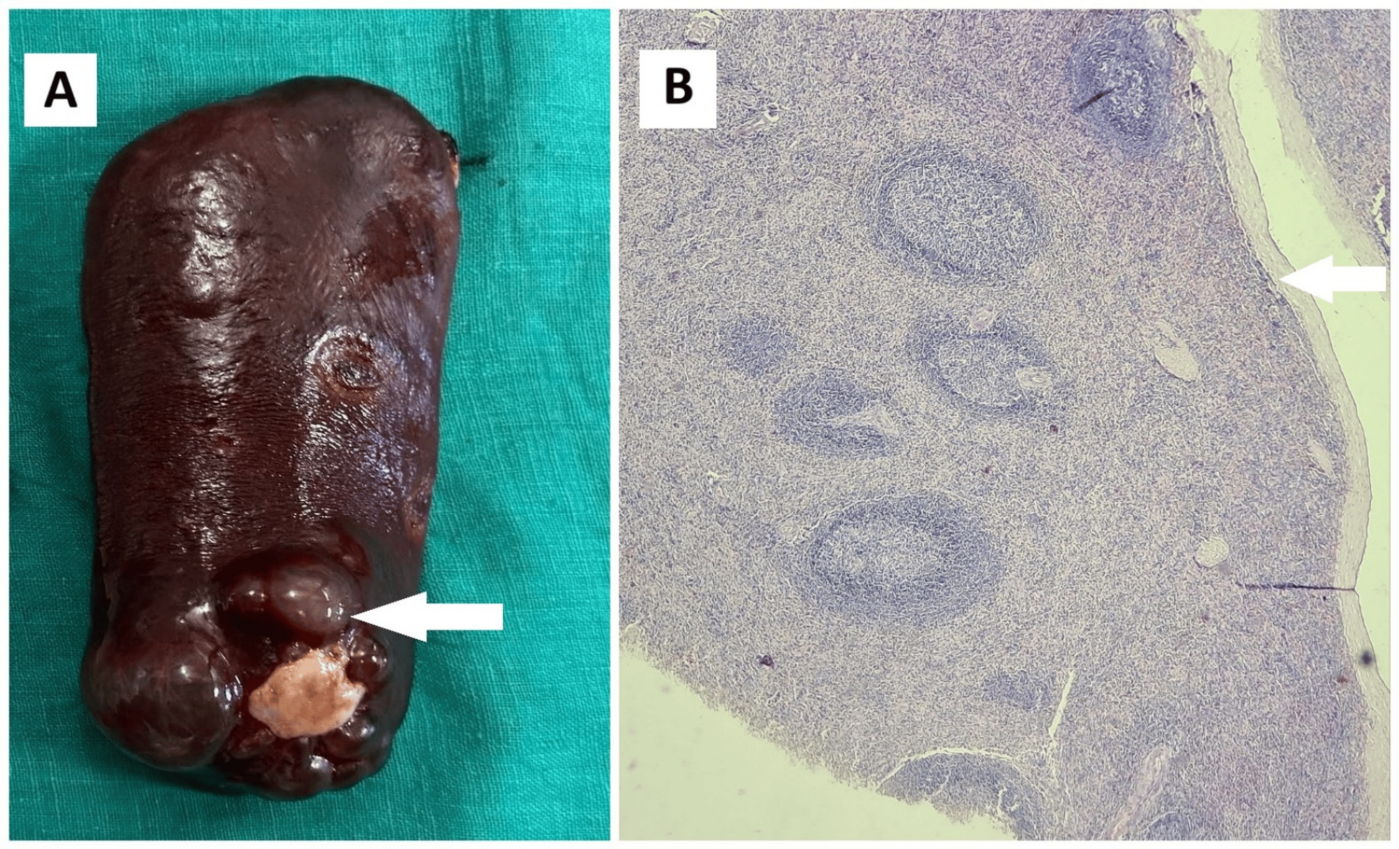
Primary splenic cyst (A) Gross specimen of the spleen with the cyst (white arrow); (B) Histopathological image showing cuboidal to columnar cyst wall lining epithelium with splenic parenchyma (white arrow).

Since she was symptomatic and in a diagnostic dilemma, she underwent elective laparoscopic splenectomy. Gross examination showed a multiloculated cystic mass (Figure 8A). 

Histopathology confirmed a primary benign epithelial splenic cyst (Figure 8B). The patient recovered uneventfully in the postoperative period. 

Case 5: Post-drainage infection of primary splenic cyst

**Figure 9:**
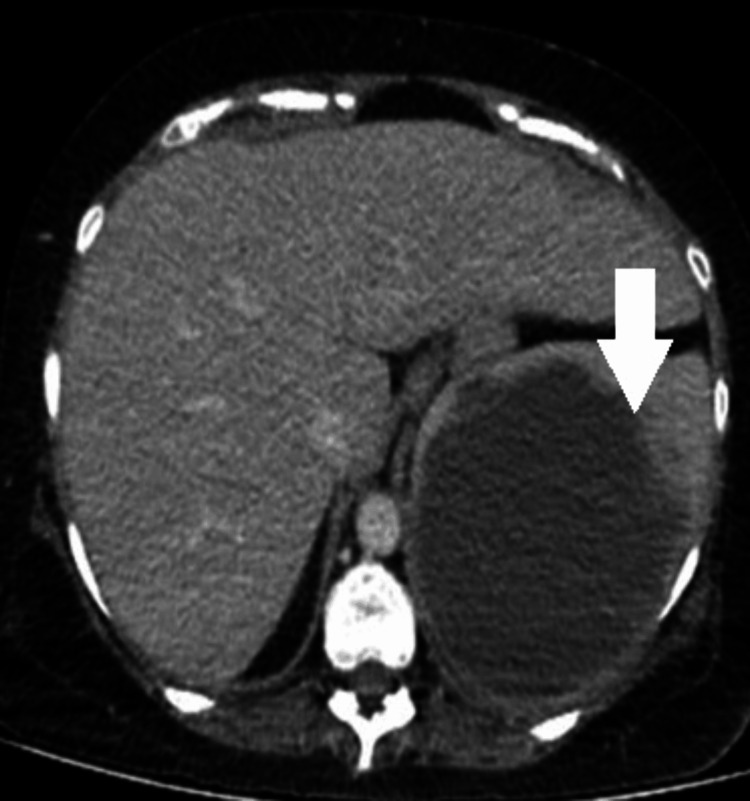
Primary splenic cyst with infection Contrast-enhanced CT abdomen showing a cystic lesion in the spleen (white arrow).

A 27-year-old female patient presented with left hypochondriac pain and intermittent fever for one week. She was already diagnosed as a case of splenic cyst for which percutaneous drainage (PCD) was done one month before this presentation in an outside hospital. CT imaging of abdomen and pelvis showed a well-defined hypodense non-enhancing cystic lesion 7.8x8 cm and calcification in the wall of superior pole of spleen (Figure 9) suggestive of splenic abscess.

**Figure 10:**
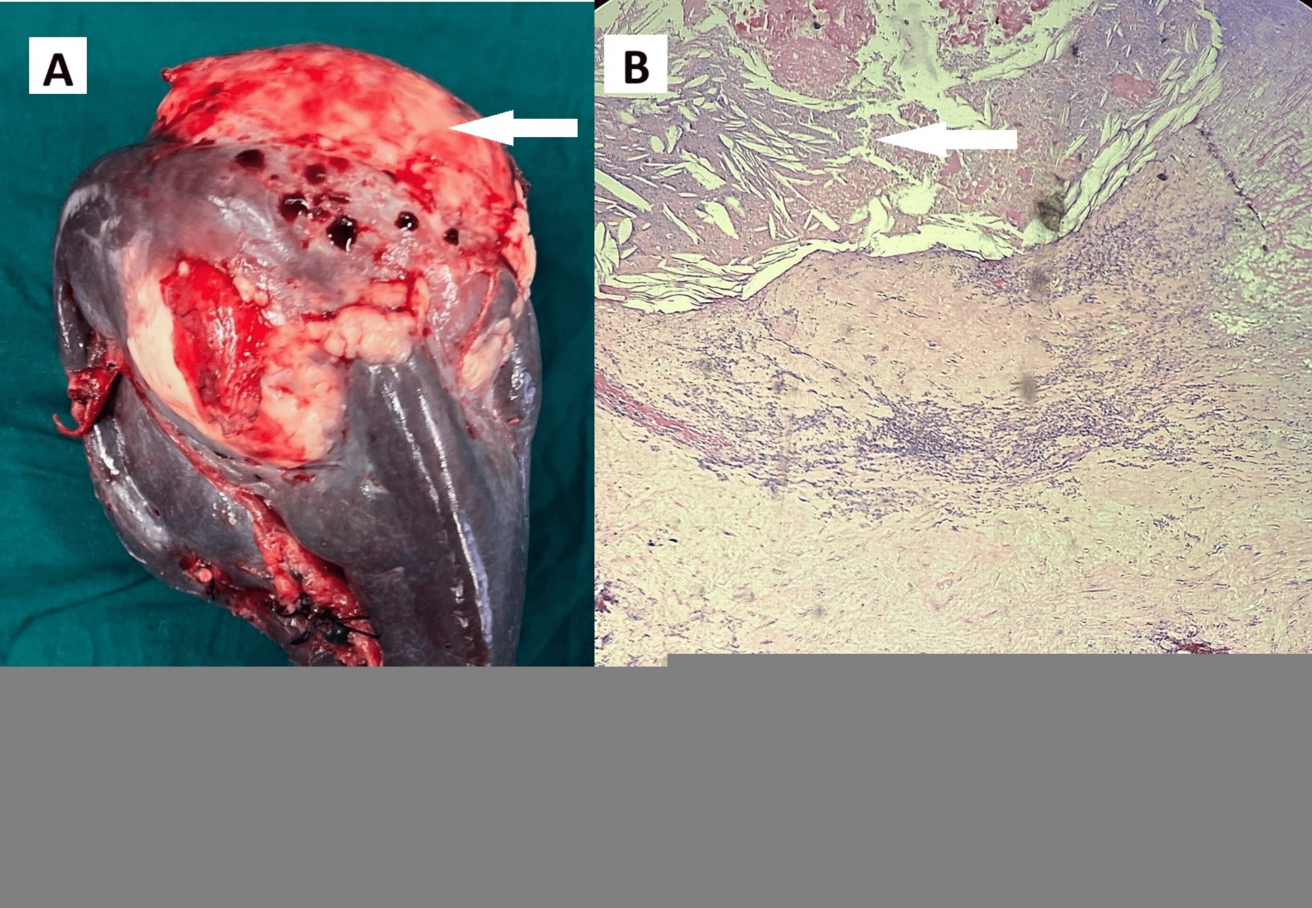
Splenic cyst with an infected complication (A) Gross specimen showing spleen with abscess cavity (white arrow); (B) Histopathological image showing the cyst wall with cholesterol clefts and inflammatory exudates (white arrow).

She was symptomatic with signs of infection in the form of leukocytosis (Table 1) which might have been introduced from an external source related to previous catheter insertion into the splenic cyst, and hence elective open splenectomy was planned and performed. Intraoperatively, dense adhesions were noted around the spleen. Figure 10A shows gross specimen of the spleen with abscess cavity.

Histopathology revealed a splenic abscess (Figure 10B).

Case 6: Primary splenic angiosarcoma with spontaneous rupture

**Figure 11:**
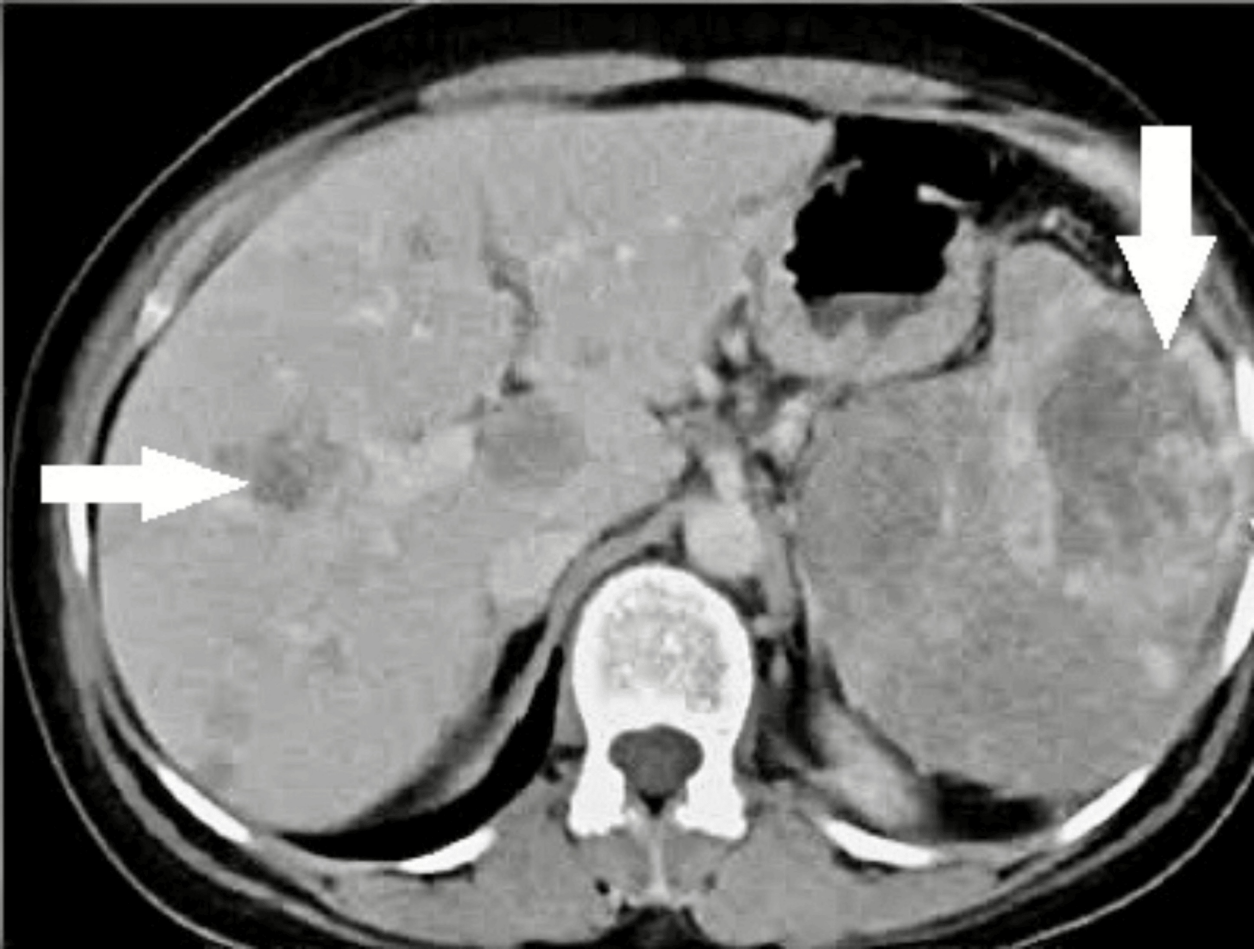
Primary splenic angiosarcoma with spontaneous rupture Contrast-enhanced CT scan showing multiple heterogeneous lesions in both liver and spleen with hemoperitoneum. The white arrows show the liver metastases and splenic lesion.

A 44-year-old female patient presented to the emergency department with sudden onset abdominal pain, hypotension, and severe anemia. CT imaging of the abdomen and pelvis revealed multiple hetero-dense lesion with internal hemorrhage noted in both lobes of the liver, with the largest measuring 6.3x6.9 cm in segment eight of the liver and also with multiple hetero-dense lesion in the spleen, with the largest measuring 5.2x5 cm, and dense free fluid was also noted (Figure 11). 

Since the patient developed signs of peritonism and hemodynamic instability, an urgent laparotomy was performed. Intraoperatively, hemoperitoneum of around 2.5 liters was noted. The spleen was grossly enlarged and friable with rupture noted near the superior pole. Multiple hyper-vascular lesions were noted in both the spleen and the liver. Figure 11 shows a metastatic lesion in the liver. Splenectomy was performed. Diffuse ooze was noted from the diaphragmatic attachment of the spleen in spite of adequate hemostatic measures. So packing was done in the splenic fossa which was removed in the second look laparotomy after 48 hrs.

**Figure 12:**
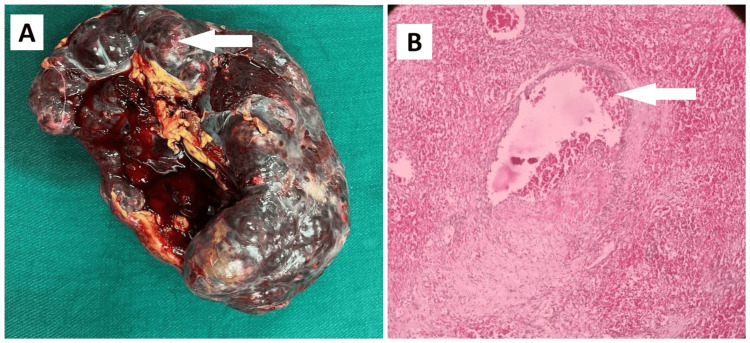
Primary splenic angiosarcoma (A) Gross specimen showing spleen with hyper vascular necrotic tumor (white arrow); (B) Histopathology image showing dilated vascular channels with intervening pleomorphic cells (white arrow).

Figure 12A shows the gross specimen, demonstrating that the entire spleen was replaced by the hyper vascular mass.

Histopathology was consistent with angiosarcoma of the spleen with strong positivity of ERG (ETS family of transcription factor expressed in endothelial cells) in tumor cells and Ki67 index of 50% (Figure 12B). She had already presented with distant metastases of the liver and bones at the time of diagnosis. Unfortunately, she succumbed to the disease on postoperative day 10.

## Discussion

Splenectomy is most often performed for trauma and hematological disorders such as immune thrombocytopenic purpura (ITP) or hereditary spherocytosis, and certain malignancies [1,2]. However, rare conditions may also necessitate splenic removal, either due to life-threatening complications or diagnostic uncertainty [3]. Our case series highlights a diverse spectrum of such uncommon but significant indications: congenital afibrinogenemia, pancreatitis-related splenic complications, primary splenic cyst, and ruptured angiosarcoma of the spleen.

Congenital afibrinogenemia is a rare bleeding disorder characterized by absent or severely low fibrinogen levels. It is an autosomal recessive disease that results from a mutation of one of the three genes (FGA, FGB, FGG) that code the three polypeptide chains of fibrinogen, disrupting its synthesis and stability, ultimately resulting in life-threatening hemorrhage [4]. While spontaneous hemorrhage is well known, visceral hemorrhage, especially with splenic involvement, is rare and potentially fatal [5,6]. Bleeding episodes in patients with afibrinogenemia are treated with fibrinogen concentrates as the first-line therapy. Cryoprecipitate and fresh frozen plasma are other options when fibrinogen concentrates are unavailable. A growing body of evidence shows that human fibrinogen concentrates are safe and effective; however, they may produce allergic or hypersensitivity reactions. These can range from mild episodes of skin rashes /urticaria to life-threatening anaphylactic reactions. We resuscitated our patient with intravenous fluids, blood and fibrinogen concentrates. But the patient was hemodynamically unstable with signs of peritonitis. In the first case, the failure of conservative measures warranted splenectomy, which proved lifesaving. Recent studies suggest liver transplantation as one of the novel options in treating congenital afibrinogenemia in future [7].

Pancreatitis is a common condition with well-documented complications. Splenic involvement in chronic pancreatitis, particularly in the form of pseudocysts, is uncommon [8]. The pancreas and the spleen are anatomical allies, and for this reason, complications involving the spleen can occur as a result of pancreatitis. The pancreatic tail, splenic hilum, its associated vessels, and the peritoneum of the anterior pancreatic surface are vital in allowing leaked proteolytic pancreatic enzymes. This causes enzymatic degradation of splenic parenchyma and opening up of red pulp with rupture of trabecular vessels, thereby causing subcapsular splenic hematoma leading to spontaneous rupture. There are case reports which have demonstrated that pancreatic pseudocysts can result in splenic penetration associated with hemorrhage and mortality [9]. Although percutaneous or endoscopic drainage may be attempted in selected cases, emergency splenectomy combined with distal pancreatectomy remains the treatment of choice when bleeding, rupture, or infection occurs [10,11].

Primary splenic cysts are uncommon, accounting for <10% of all non-parasitic splenic cysts. They are typically true cysts, meaning they are lined by epithelium and are distinct from secondary (pseudocysts) which usually occur after trauma or inflammation and lack an epithelial lining. Primary splenic cysts can be either congenital (epithelial/epidermoid) or neoplastic (hemangioma/lymphangioma), or parasitic (echinococcal) [12]. They may present with abdominal discomfort or be complicated by infection, hemorrhage, or rupture. Congenital epithelial splenic cysts are often discovered incidentally and if symptomatic usually warrant surgical intervention (splenectomy) [13,14]. In our cases, splenectomy provided both a definitive diagnosis and relief from compressive symptoms. Most epithelial cysts are benign, and elective laparoscopic splenectomy is a safe approach when indicated.

Primary splenic angiosarcoma is an extremely rare and aggressive malignancy with <200 cases reported globally [15]. It most often presents in the sixth decade but may occur earlier. It commonly presents with nonspecific symptoms or as a spontaneous splenic rupture, often leading to delayed diagnosis [16,17]. Metastases to the liver, lungs, or bones are frequently present at diagnosis and the prognosis is usually poor. While splenectomy is essential for bleeding control and histological diagnosis, overall survival remains limited. Diagnosis requires histopathologic evaluation, with immunohistochemical markers such as CD31, CD34, and ERG confirming endothelial origin [18]. Despite surgical resection, prognosis remains poor, with median survival of less than 12 months due to early metastasis and tumor aggression. In the sixth case, the patient underwent emergency surgery but succumbed in the early postoperative period and developed widespread metastases, reflecting the tumor’s aggressive biology.

In our series, splenectomy served several important purposes: hemorrhage control, removal of infected or ruptured cysts, and definitive diagnosis in uncertain or malignant cases. Each case reflects the utility of splenectomy beyond conventional indications and highlights the necessity of early surgical intervention when conservative treatment fails or imaging findings are concerning. Furthermore, histopathology proved essential in confirming the underlying pathology, especially in distinguishing between benign cysts and neoplastic processes.

A high index of suspicion, appropriate imaging, and timely surgical consultation are key in managing these rare splenic conditions. In selected patients, a multidisciplinary team including surgeons, hematologists, gastroenterologists, and radiologists can significantly improve outcomes.

## Conclusions

Although splenectomy is most commonly performed for well-established indications such as trauma, hematologic disorders, and malignant involvement of the spleen, this case series highlights the indispensable role of splenectomy in a variety of rare, atypical, and diagnostically challenging conditions. Each case presented demonstrates how splenectomy served either as a life-saving emergency procedure or as a definitive diagnostic and therapeutic intervention when conservative or non-surgical measures failed.

Congenital afibrinogenemia with spontaneous splenic rupture illustrates how inherited bleeding disorders, although rare, can present with life-threatening visceral hemorrhage requiring immediate surgical management. In the context of chronic pancreatitis, splenic pseudocysts represent an uncommon but serious complication that can lead to spontaneous rupture and massive hemorrhage, necessitating prompt surgical intervention, often in combination with distal pancreatectomy. Primary splenic cysts, while typically benign, can become symptomatic, become infected following drainage, or mimic parasitic or neoplastic lesions radiologically, further justifying splenectomy for definitive management. Splenic angiosarcoma represents an extreme example of an aggressive vascular malignancy that frequently presents with spontaneous rupture and advanced metastasis. In such cases, splenectomy may offer temporary hemodynamic stabilization and histologic confirmation, although long-term prognosis remains poor.

This series emphasizes the critical importance of maintaining a broad differential diagnosis when evaluating splenic pathology, particularly in the setting of atypical clinical or radiologic features. Early surgical consultation should be considered when patients present with signs of hemodynamic compromise, abdominal pain of unclear origin, or when imaging suggests cystic, vascular, or hemorrhagic lesions of the spleen. Moreover, the intraoperative and histopathological findings from splenectomy often provide diagnostic clarity in cases where non-invasive methods are inconclusive. Ultimately, timely recognition of these rare but significant conditions and an appropriately tailored surgical approach can result in improved patient outcomes. Multidisciplinary collaboration between surgeons, radiologists, hematologists, and pathologists is essential in managing such complex presentations. While the spleen is often considered expendable in surgical practice, its diseases-especially the rare ones-can challenge even experienced clinicians. Splenectomy, when appropriately indicated, remains a powerful tool in the diagnostic and therapeutic armamentarium of surgeons managing complex abdominal conditions.
